# A mixed-methods systematic review protocol to examine the use of physical restraint with critically ill adults and strategies for minimizing their use

**DOI:** 10.1186/s13643-016-0372-8

**Published:** 2016-11-21

**Authors:** Louise Rose, Craig Dale, Orla M. Smith, Lisa Burry, Glenn Enright, Dean Fergusson, Samir Sinha, Lesley Wiesenfeld, Tasnim Sinuff, Sangeeta Mehta

**Affiliations:** 1 Department of Critical Care Medicine, Sunnybrook Health Sciences Centre, 2075 Bayview Ave, Toronto, M4N 3M5 Canada; 2 Lawrence S. Bloomberg Faculty of Nursing, University of Toronto, 155 College St., Toronto, M5T 1P8 Canada; 3 Institute for Clinical Evaluative Sciences, Veterans Hill Trail, 2075 Bayview Avenue G1 06, Toronto, M4N 3M5 Canada; 4 Provincial Centre of Weaning Excellence, Michael Garron Hospital, 825 Coxwell Ave, East York, M4C 3E7 Canada; 5 Department of Critical Care, Nursing/Clinical Research, Li Ka Shing Knowledge Institute, St. Michael’s Hospital, 30 Bond St, Toronto, ON M5B 1W8 Canada; 6 Leslie Dan Faculty of Pharmacy, University of Toronto, 144 College St, Toronto, M5S 3M2 Canada; 7 Mount Sinai Hospital, 600 University Ave, Toronto, M5G 1X5 Canada; 8 Clinical Epidemiology Program, Ottawa Hospital Research Institute, 500 Smyth Road, Ottawa, ON K1H 8L6 Canada; 9 Faculty of Medicine, University of Ottawa, 451 Smyth Rd, Ottawa, K1H 8M5 Canada; 10 Faculty of Medicine, University of Toronto Medical Sciences Building, 1 King’s College Cir #3172, Toronto, M5S 1A8 Canada; 11 Department of Family and Community Medicine, 500 University Ave, Toronto, M5G 1V7 Canada; 12 Institute of Health Policy, Management and Evaluation, University of Toronto, 155 College St, Toronto, ON M5T 3M6 Canada

**Keywords:** Physical restraint, Patient safety, Intensive care, Mechanical ventilation, Systematic review

## Abstract

**Background:**

Critically ill patients frequently experience severe agitation placing them at risk of harm. Physical restraint is common in intensive care units (ICUs) for clinician concerns about safety. However, physical restraint may not prevent medical device removal and has been associated with negative physical and psychological consequences. While professional society guidelines, legislation, and accreditation standards recommend physical restraint minimization, guidelines for critically ill patients are over a decade old, with recommendations that are non-specific. Our systematic review will synthesize evidence on physical restraint in critically ill adults with the primary objective of identifying effective minimization strategies.

**Methods:**

Two authors will independently search from inception to July 2016 the following: Ovid MEDLINE, CINAHL, Embase, Web of Science, Cochrane Library, PROSPERO, Joanna Briggs Institute, grey literature, professional society websites, and the International Clinical Trials Registry Platform. We will include quantitative and qualitative study designs, clinical practice guidelines, policy documents, and professional society recommendations relevant to physical restraint of critically ill adults. Authors will independently perform data extraction in duplicate and complete risk of bias and quality assessment using recommended tools. We will assess evidence quality for quantitative studies using the Grading of Recommendations Assessment, Development and Evaluation (GRADE) approach and for qualitative studies using the Confidence in the Evidence from Reviews of Qualitative Research (CERQual) guidelines. Outcomes of interest include (1) efficacy/effectiveness of physical restraint minimization strategies; (2) adverse events (unintentional device removal, psychological impact, physical injury) and associated benefits including harm prevention; (3) ICU outcomes (ventilation duration, length of stay, and mortality); (4) prevalence, incidence, patterns of use including patient and treatment characteristics and chemical restraint; (5) barriers and facilitators to minimization; (6) patient, family, and healthcare professional perspectives; (7) professional society-endorsed recommendations; and (8) evidence gaps and research priorities.

**Discussion:**

We will use our systematic review findings to produce updated guidelines on physical restraint use for critically ill adults and to develop a professional society-endorsed position statement. This will foster patient and clinician safety by providing clinicians, administrators, and policy makers with a tool to promote minimal and safe use of physical restraint for critically ill adults.

**Systematic review registration:**

PROSPERO CRD42015027860

**Electronic supplementary material:**

The online version of this article (doi:10.1186/s13643-016-0372-8) contains supplementary material, which is available to authorized users.

## Background

Physical restraint is highly prevalent in the critically ill, with up to 75% of mechanically ventilated adults having physical restraints applied at least once during their intensive care unit (ICU) admission [1]. Annually, an estimated 13 million people are admitted to ICUs worldwide [2] with approximately 30% requiring mechanical ventilation [3, 4]. While acknowledging variation in international physical restraint practice [5], some reports indicate the prevalence of physical restraint is greater than 70% of mechanically ventilated critically ill patients [6] who are in turn at risk of associated adverse physical and psychological consequences annually. While government legislation, hospital accreditation standards, and professional society guidelines recommend physical restraint minimization [7], available guidelines on alternative strategies to physical restraint for critically ill adults [8] are outdated and are not specific regarding recommendations to support the minimization of physical restraint use.

In the ICU, physical restraint is frequently used for clinician concerns for patient and/or clinician safety including prevention of accidental removal of medical devices (e.g. endotracheal tubes, central venous catheters) [5]. However, evidence does not uniformly or persuasively indicate that physical restraint effectively reduces the risk of accidental device removal [9–11]. For example, a multi-centre study conducted in 39 hospitals in the USA found that 44% of hospitalized patients were restrained at the time of device removal [12] and multiple studies of critically ill patients indicate they were restrained at the time of self-extubation or other device removal [10–15]. A recent case-control study with matching by mechanical ventilation duration further reported that physically restrained patients were in fact five times more likely to experience unplanned extubation [16].

Despite the intention to promote patient and clinician safety, physical restraint can adversely impact patient safety resulting in detrimental physical and psychological consequences [17–22]. Negative physical consequences include tissue injury [23], immobility, pressure ulcers, and nosocomial infection [24]. Negative psychological consequences include agitation, disorientation, psychological trauma, and delirium, a serious and potentially fatal complication of critical illness [18–20]. Physical restraint without sedation has been associated with delusional memories and post-traumatic stress disorder in ICU survivors [21, 22]. Finally, physical restraint is often not removed expeditiously when agitation has resolved, thereby negatively impacting patient safety.

Given the recognized adverse physical and psychological consequences of physical restraint and the lack of efficacy for preventing device removal, government legislation, hospital accreditation standards, and professional society guidelines *all* advocate for minimization of physical restraint across all healthcare settings [8, 25, 26]. However, these recommendations lack specificity on how to do this, resulting in institutional and healthcare team practice variation with respect to triggers for physical restraint utilization and removal, use of alternatives, documentation standards, and patient monitoring during physical restraint.

The identification of effective physical restraint minimization strategies is an immediate clinical and research imperative and has implications in terms of patient- and family-reported experience and outcome measures. To address this need, we will conduct a systematic review with the overall objective of producing an updated practice guideline on physical restraint for critically ill adults and developing a professional society-endorsed position statement. Our objectives include synthesizing quantitative studies of the (1) efficacy and effectiveness of physical restraint minimization strategies; (2) adverse events (unintentional device removal, psychological impact, physical injury) and associated benefits including harm prevention; (3) ICU outcomes (ventilation duration, length of stay, and mortality); and (4) prevalence, incidence, and patterns of use including patient and treatment characteristics and chemical restraint. Additionally, we will synthesize quantitative and qualitative evidence on barriers and facilitators to minimization and patient, family member, and ICU healthcare professional perspectives and identify professional society-endorsed recommendations relevant to ICU patients and evidence gaps and research priorities.

## Methods

This review protocol was prepared using the Preferred Reporting Items for Systematic Reviews and Meta-Analyses Protocol (PRISMA-P) guidelines [27]. We completed the PRISMA-P checklist (Additional file 1). We registered the protocol on International Prospective Register of Systematic Reviews (PROSPERO) (CRD42015027860).

### Data sources and search strategy

We created a preliminary search strategy (Additional file 2) as an iterative process under the guidance of an experienced information specialist. For the purposes of this systematic review and guideline update, we have defined physical restraint as mechanical devices that restrict patients’ movements [8]. Prior to execution of the search, a second information specialist used the Peer Review for Electronic Search Strategies (PRESS) template [28, 29] to review the search strategy. We will search the following electronic databases from inception to July 2016: Ovid MEDLINE® (OvidSP), Ovid MEDLINE® In-Process & Other Non-Indexed Citations, CINAHL (EBSCOhost), Embase (OvidSP), and ISI Web of Science and Conference Proceedings. We will search for systematic reviews within the Cochrane Library, PROSPERO, and the Joanna Briggs Institute and will search for unpublished studies and ongoing trials on the http://apps.who.int/trialsearch website. We will search major guideline sites (e.g. CMA Infobase, National Guideline Clearinghouse) and websites of relevant professional societies and conduct general searches using tools such as Google Scholar, ScienceWatch, and MSN. We will examine reference lists of relevant studies and will contact corresponding authors for additional published or unpublished work.

Search strategies will utilize a combination of controlled vocabulary (e.g. ‘Intensive Care Units’, ‘Critical Care’, ‘Restraint, Physical’) and keywords (e.g. ICU, high-dependency unit, restraint). Vocabulary and syntax will be adjusted across databases. We will remove animal-only studies and opinion pieces (e.g. editorials, letters). We will not impose language restrictions in our database searches but will only seek to obtain policy documents written in English. As we are including a range of study designs, we will not apply study-specific filters.

### Study eligibility criteria

We will include a range of study designs, including qualitative studies describing perspectives/restraint experience as well as clinical practice guidelines, policy documents, and professional society recommendations relevant to critically ill adults. To determine the efficacy/effectiveness and safety of physical restraint minimization strategies, we will include parallel group randomized controlled trials including crossover trials, quasi-randomized trials (such as alternate allocation based on day of the week), and non-randomized studies including non-randomized controlled trials; controlled before and after studies; historically controlled studies; and retrospective or prospective cohort studies that include a control group. Inclusion of evidence from qualitative studies that explore the experience of patients and family members receiving interventions and healthcare providers delivering the intervention, as well as studies that evaluate barriers and facilitators to implementation of interventions, have an important role in maximizing the value of a systematic review to inform clinical practice, policy, and decision-making [30–32].

#### Population

Our population of interest is adults admitted to a high intensity care setting including ICUs, specialized weaning centres, and high-dependency units. We will include all high intensity care setting patient populations including those admitted for medical, surgical, neurological, cardiac, and trauma diagnoses. We will exclude studies of physical restraint use in patients admitted to other acute care settings such as the emergency department, postoperative recovery units, hospital floors/wards, psychiatry, and in long-term care settings.

#### Intervention

For studies examining the efficacy and safety of physical restraint minimization, interventions will include any strategy used for the purpose of decreasing the frequency and duration of physical restraint. Such strategies may comprise pharmacological and non-pharmacological interventions used to alleviate anxiety and optimize analgesia and alternatives to ensure patient safety such as sitters or observers. Patients may receive chemical restraint in the form of sedatives and neuromuscular blockers. For non-interventional and qualitative studies, we will include data from patients that are not physically restrained during admission to a high care intensity setting.

#### Comparators

For studies examining the efficacy and safety of physical restraint minimization and non-interventional and qualitative studies, comparator groups will receive usual care that comprises physical restraint methods such as wrist restraints, ankle restraints, chest belt, bed rails, vest restraints, mitts or mittens, four-point restraints, and five-point restraints.

#### Outcomes

Our primary outcome of interest is the efficacy/effectiveness of physical restraint minimization strategies defined as the proportion of patients physically restrained. Secondary outcomes include (1) adverse events including unintentional device removal (endotracheal and tracheostomy tubes, catheters, intravenous lines, feeding tubes, and wound drains); delirium; severe agitation; psychological impact including anxiety, depression, and post-traumatic stress disorder (quantitative measures or qualitative description); physical injury resulting from restraint; and associated benefits including harm prevention; (2) ICU outcomes including duration of mechanical ventilation, ICU length of stay, and mortality; (3) prevalence, incidence, and patterns of physical restraint use including patient and treatment characteristics and chemical restraint; (4) barriers and facilitators to minimization; and (5) patient, family member, and healthcare professional perspectives on physical restraint. Additionally, we will identify recommendations for restraint minimization endorsed by professional societies and accreditation and regulatory agencies and evidence gaps and priorities for research.

### Screening and data extraction

Two authors (LR, GE) will independently screen search results against eligibility criteria. Full-text publications of all potentially relevant articles, selected by either author, will be retrieved and examined for eligibility. We will resolve any disagreements though discussion and refer to an independent arbiter (CD) if required. We will use the reference management software EndNote X7 [33] to remove duplicates and sort exclusions and inclusions using the create group function. We will document the search strategy and study selection process using a PRISMA flow diagram [34].

Teams of two authors will independently extract data from selected studies on key components addressing our research questions including features of study design, patient characteristics, prevalence and incidence, study outcomes, benefits and adverse events, perceptions, and recommendations using designed data extraction forms, iteratively refined based on pilot data extraction. We have developed three data extraction forms for (1) studies with a control group, (2) studies without a control group, and (3) studies with a qualitative design. Due to inherent difficulties with determining what constitute findings in a qualitative research [35], we will extract category- or theme-level evidence from the findings or results section of the included papers only. Extraction will be checked and any disagreement resolved by a third author. We will confirm all data extraction, and where necessary, discrepancies will be resolved by an independent arbiter (LR).

### Risk of bias and quality assessment

We will critically appraise included papers in duplicate and independently for risk of bias. For randomized and quasi-randomized trials, we will use the domain-based evaluation recommended by The Cochrane Handbook. [36] These domains include the following: random sequence generation, allocation concealment, blinding, incomplete outcome data, selective reporting, and other biases. For each domain, we will assign a judgement regarding the risk of bias as ‘high risk of bias’, ‘low risk of bias’, or ‘unclear risk of bias’ [36]. Once we achieve consensus on the quality assessment of the six domains for eligible studies, we will assign them to the following categories: low risk of bias: low risk scored in all domains; high risk of bias: high risk scored in one or more domains; and unclear risk of bias: unclear risk scored in one or more domains (and no domain at the high risk of bias). For quality assessment of observational studies, we will use the SIGN checklists for cohort and case-control studies as recommended by the Quality Assessment Tools Project Report [37]. We will construct a ‘risk of bias’ table to present the results.

For qualitative studies, we will assess study quality using the multi-dimensional concept of quality recommended by the Cochrane Quality and Intervention Methods Group [38] that includes (1) quality of reporting, e.g. explicitness in reporting all study aspects; (2) methodological rigour, e.g. validity and reliability of study design and process; and (3) overall conceptual depth and breadth, e.g. if stated study aims, rationale, or theory (if the study is explicitly theoretically informed) are reflected in the study design, process, and findings. Two authors will independently appraise study quality using the 10 questions of the 2014 Critical Appraisal Skills Programme (CASP) quality assessment tool for qualitative studies [39]. Each of these 10 questions will be answered as ‘yes’, ‘no’, or ‘unclear’. The CASP tool does not consider the more conceptual or theoretical aspects of qualitative studies; the same two authors will independently appraise study quality using the seven criteria outlined by Popay et al. [40]. These seven criteria include (1) illumination of subjective meaning, (2) adaptation and responsiveness of research design, (3) sample provides appropriate knowledge, (4) description sufficiently detailed, (5) sources of knowledge compared and contrasted, (6) shift from description to analysis and interpretation, and (7) claims for generalizability. We will present quality assessments in tabular format.

### Approach to evidence synthesis

Search results will be summarized in a Preferred Reporting Items for Systematic Reviews and Meta-Analyses (PRISMA) study flow diagram [41]. We will categorize studies according to study design and then summarize study characteristics within these categories using frequencies and percentages for categorical variables and means and standard deviations or median and interquartile ranges for continuous variables depending on data distribution. We will generate tables reporting types and frequency of adverse outcomes associated with physical restraint, benefits associated with physical restraint, prevalence and incidence overall and according to country, characteristics of physically restrained patients, and strategies used to minimize physical restraint. We anticipate qualitative data will describe patient, family, and healthcare provider perspectives on physical restraint use as well as barriers and facilitators to physical restraint minimization. We will generate tables of author-reported categories, themes, and subthemes as well as existing professional society recommendations related to physical restraint use. We will undertake thematic content analysis [42] to identify common categories and themes within these categories. We will similarly examine professional society guidelines and recommendations to identify commonalities across guidelines.

### Measurement of treatment effect

If we identify sufficient studies evaluating interventions to minimize physical restraint, we will perform meta-analyses for the following outcomes: proportion of patients physically restrained, duration of mechanical ventilation, ICU length of stay, mortality, and adverse events. If we identify fewer than three randomized or quasi-randomized trials, we will include non-randomized studies in this meta-analysis.

For binary outcomes, we will calculate risk ratios (RRs) with 95% confidence intervals (CIs) using a random effects model. For continuous variables, we will calculate a pooled difference of means with 95% confidence intervals using a DerSimonian Laird random effects model. If the mean (standard deviation, SD) is not reported or unavailable from the study authors, we will use the median (IQR) to approximate the mean using the method described by Hozo and colleagues [43] and will calculate an approximate SD from the IQR [36]. If required, we will log transform skewed data. We will consider a two-sided *P* value <0.05 to be significant. We will use RevMan 5.3 software to conduct meta-analyses. If we identify insufficient studies for meta-analyses, we will provide a descriptive qualitative synthesis.

### Subgroup and sensitivity analysis

If we identify sufficient studies of interventions to minimize physical restraint, we will perform subgroup analyses considering intervention type and patient characteristics such as age and admission category (medical, surgical, cardiac, neurological, trauma). We will conduct two sensitivity analyses, one excluding non-randomized studies and the second excluding studies rated as high risk of bias.

### Unit of analysis issues

We will use individual study participants as the unit of analysis. If we identify multi-arm studies, we will combine groups to create a single pairwise comparison as recommended by the Cochrane Handbook for Systematic Reviews of Interventions [36]. If combining groups is not possible or feasible, we will select only one treatment group receiving the most intense level of intervention to minimize physical restraint and a control group receiving the least intervention from each study.

### Dealing with missing data

We (LR) will attempt to contact study authors for unreported data or clarification of study methods using a maximum of three e-mails. If data remains unavailable, we will analyse the available data and report the potential impact of missing data in the discussion section.

### Assessment of heterogeneity

If we identify sufficient studies evaluating interventions to minimize physical restraint, we will assess clinical and methodological heterogeneity with forest plots and chi-square tests (*P* < 0.05 represents significant heterogeneity) and using the *I* ^2^ statistic, which represents the percentage of variability across studies attributable to heterogeneity rather than chance [36]. If the *I* ^2^ statistic is >50% statistic, indicating moderate to substantial heterogeneity, we will assess the type and sources of heterogeneity (clinical and methodological) such as ICU patient type and physical restraint minimization intervention used.

### Publication bias

If sufficient studies are identified, we will assess for publication bias by constructing a funnel plot of all studies included in any analysis [44] as well as using Egger’s regression test [45] and the Macaskill test [46, 47].

### Assessing confidence in evidence

We will use the Grading of Recommendations Assessment, Development and Evaluation (GRADE) approach [48] to assess the confidence in the evidence of effectiveness arising from studies evaluating interventions to minimize physical restraint for the following outcomes: proportion of patients physically restrained, duration of mechanical ventilation, ICU length of stay, mortality, and adverse events. We will present our GRADE assessments in a summary of findings table.

We will use the Confidence in the Evidence from Reviews of Qualitative Research (CERQual) approach to assess confidence in qualitative evidence synthesis [49]. This relatively new approach makes judgements on four components: (1) methodological limitations of included studies, (2) relevance of contributing studies to the research question, (3) coherence of study findings, and (4) adequacy of the data supporting the study findings. Judgements related to the four CERQual components will be summarized in a CERQual Qualitative Evidence Profile (Additional file 3) [49]. Two authors will independently assess each CERQual component individually and across the four components to make a final assessment. We will rate overall assessment of confidence as high, moderate, low, or very low and provide a reason for this judgement. We will assign high confidence if it is *highly likely*, moderate confidence if *likely*, low confidence if it is *possible*, and very low confidence if it is *not clear* that the review finding is a reasonable representation of the phenomenon of interest [49]. We will present our CERQual findings in a summary of qualitative findings table.

Tabulated data, quality of evidence, and confidence in evidence assessment findings will be provided to an interprofessional and international guideline development group to develop recommendations for inclusion in an updated practice guideline on physical restraint for critically ill adults.

## Discussion

While nursing homes and psychiatric units have seen a significant reduction in physical restraint use over the last two decades, use amongst the critically ill remains extremely common and may have detrimental physical and psychological consequences [12, 17–22]. Data from a cohort of hospitals in the USA indicate that over 2 years, approximately 27,000 patients were physically restrained daily, with most physical restraint use (56%) occurring in ICUs, despite this care location accounting for only 16% of overall hospital days [50]. In a 2013 survey of 121 French ICUs, physical restraints were used at least once in more than 50% of mechanically ventilated patients. Moreover, ventilated patients in most ICUs (65%) were restrained for greater than half the ventilation duration [51]. Interestingly, practice variation exists with some countries declaring minimal to no physical restraint use in the ICU [5]. In a binational comparison study, physical restraint prevalence in a sample of three US ICUs was 39%, compared with 0% in two Norwegian ICUs, though Norwegian patients were more sedated [52]. Therefore, physical restraint is highly prevalent in the critically ill patient population with some evidence of adverse consequences for patients. As such, identification of effective physical restraint minimization strategies is an immediate clinical and research imperative with the potential to influence patient- and family-reported experience and outcomes.

We will use our mixed-methods systematic review findings to develop a professional society-endorsed position statement and to update the 2003 guidelines [8] on physical restraint for critically ill adults. We will engage key stakeholders and knowledge users including ICU survivors and family members in the development of these documents and to inform our knowledge translation strategies. We will also engage these individuals in the identification of research gaps and priorities for research in relation to physical restraint of critically ill patients. We anticipate our systematic review and subsequent guideline development will foster patient and clinician safety by providing clinicians, administrators, and policymakers with a practical and evidence-based tool to promote minimal and safe use of physical restraint for critically ill adults.

## Additional files

Additional file 1: PRISMA-P checklist. PRISMA-P checklist. (DOCX 35 kb)
Additional file 2: Search strategies_MEDLINE. Medline search strategy. (DOCX 14 kb)
Additional file 3: CERQual Qualitative Evidence Profile. CERQual Qualitative Evidence Profile. (DOCX 14 kb)

## Abbreviations

CASP: Critical Appraisal Skills Programme
CERQual: Confidence in the Evidence from Reviews of Qualitative Research
CI: confidence interval
GRADE: Grading of Recommendations Assessment, Development and Evaluation
ICU: Intensive care unit
PRESS: Peer Review for Electronic Search Strategies
PRISMA: Preferred Reporting Items for Systematic Reviews and Meta-Analyses
RR: risk ratio
SD: standrad deviation
SIGN: Scottish Intercollegiate Guidelines Network
US: United States
