# Supplementary material for: A mixed-methods systematic review protocol to examine the use of physical restraint with critically ill adults and strategies for minimizing their use
Source: Syst Rev. 2016 Nov 21;5:194. doi: 10.1186/s13643-016-0372-8 (PMC5117692; doi:10.1186/s13643-016-0372-8)
Supplement: Additional file 2: — Search strategies_MEDLINE. Medline search strategy. (DOCX 14 kb) [file 13643_2016_372_MOESM2_ESM.docx]

Additional File 2

MEDLINE Strategy

--------------------------------------------------------------------------------

1 Intensive Care Units/

2 Burn Units/

3 Coronary Care Units/

4 Respiratory Care Units/

5 exp Critical Care/

6 ((intensive or critical or acute) adj3 care).tw,kw.

7 (ICU or ICUs or SICU or SICUs or CCU or CCUs).tw,kw.

8 (burn$1 adj3 (unit$1 or centre$1 or center$1)).tw,kw.

9 ((cardiac or coronary or heart) adj3 (unit$1 or centre$1 or center$1)).tw,kw.

10 (respiratory adj3 (unit$1 or centre$1 or center$1)).tw,kw.

11 ((surgical or surger*) adj3 (unit$1 or centre$1 or center$1)).tw,kw.

12 Postoperative Care/

13 Postoperative Complications/

14 (postoperati* or post-operati* or postsurg* or post-surg*).tw,kw.

15 Critical Illness/

16 (critical* adj (ill or illness*)).tw,kw.

17 (high dependency adj3 (unit$1 or centre$1 or center$1)).tw,kw.

18 ((stepdown or step-down) adj3 (unit$1 or centre$1 or center$1)).tw,kw.

19 (HDU or SDU or EDSDU).tw,kw.

20 (speciali#ed weaning adj3 (unit$1 or centre$1 or center$1)).tw,kw.

21 or/1-20

22 Restraint, Physical/

23 restrain*.tw,kw.

24 ((disallow* or limit* or restrict* or stop*) adj3 movement*).tw,kw.

25 ((ankle or ankles or appendage$1 or arm or arms or body or bodies or foot or feet or hand or hands or leg or legs or limb$1 or patient$1 or wrist or wrists) adj3 tie$1).tw,kw.

26 ((belt* or strap* or tie$1) adj3 (bed or beds or down)).tw,kw.

27 (chest adj (belt* or strap* or tie*)).tw,kw.

28 (vest jacket* or vestjacket* or straight jacket* or straightjacket*).tw,kw.

29 mitten$1.tw,kw.

30 Posy belt$1.tw,kw.

31 (bedrail* or bed adj rail*).tw,kw.

31 or/22-30

32 21 and 31

33 exp Animals/ not (exp Animals/ and Humans/)

34 32 not 33

***************************
