# Supplementary material for: A mixed-methods systematic review protocol to examine the use of physical restraint with critically ill adults and strategies for minimizing their use
Source: Syst Rev. 2016 Nov 21;5:194. doi: 10.1186/s13643-016-0372-8 (PMC5117692; doi:10.1186/s13643-016-0372-8)
Supplement: Additional file 3: — CERQual Qualitative Evidence Profile. CERQual Qualitative Evidence Profile. (DOCX 14 kb) [file 13643_2016_372_MOESM3_ESM.docx]

Additional File 3: CERQual Qualitative Evidence Profile

| Objective | | | | | | | |
| --- | --- | --- | --- | --- | --- | --- | --- |
| Perspective | | | | | | | |
| Review finding | Studies contributing to review finding | Assessment of methodological limitations | Assessment of relevance | Assessment of coherence | Assessment of adequacy | Overall CERQual assessment of confidence | Explanation of judgement |
|  |  |  |  |  |  |  |  |
|  |  |  |  |  |  |  |  |
